# Aberrant expression of GBP5 in tumor-infiltrating immune cells as an onco-immunological biomarker in pan-cancer

**DOI:** 10.1007/s12672-026-05305-5

**Published:** 2026-05-27

**Authors:** Rui Liu, Jiaqian Gong, Yuehang Zhang, Qiuling Chen, Ting Han, Wenrui Li, Hanrong Zhang, Yichen Li

**Affiliations:** School of Nursing and Health, Nanfang College Guangzhou, No. 882, Wenquan Avenue, Conghua District, Guangzhou, 510970 Guangdong China

**Keywords:** Guanylate binding protein, Interferon, Tumor Microenvironment, Tumor Infiltrating Immune Cells

## Abstract

**Background:**

Guanylate binding protein 5 (GBP5) belongs to an interferon (IFN)-inducible subfamily of guanosine triphosphatases (GTPases). GBP5 has been shown to promote NLRP3 inflammasome assembly, thereby playing a key role in innate immunity and inflammation. However, the role of GBP5 in the development of various cancers and its potential as a biomarker in cancer prognosis are still unexplored.

**Methods:**

In this study, we used integrative analysis of multi-omics data, including global gene expression, tumor immune infiltration, biological functions, molecular signatures, diagnostic value of biomarkers and responses to therapies in pan-cancer to evaluate the potential of GBP5 as a biomarker in cancer prognosis.

**Results:**

Here, it is found that GBP5 is aberrantly expressed in the infiltrated immune cells in most cancer types. Furthermore, GBP5 not only responds to external cytokines, but also controls the expression of chemokines and immunomodulators. Consistently, GBP5 expression is positively associated with infiltration levels of immune cells in diverse cancer types. Moreover, GBP5 exhibits robust predictive power for response outcomes of immune checkpoint blockade (ICB) therapy compared to other 21 established biomarkers.

**Conclusion:**

Altogether, our study strongly suggests that GBP5 is an onco-immunological biomarker with potential value for cancer prognosis and the efficacy prediction of ICB immunotherapy.

**Supplementary Information:**

The online version contains supplementary material available at 10.1007/s12672-026-05305-5.

## Introduction

The tumor microenvironment (TME) is a diverse ecological niche which consists of various immune cells, stromal cells, tumor cells and the factors that they secrete. This complexity results in a chronic inflammatory, immunosuppressive and proangiogenic intratumoural environment affecting patient outcomes and treatment efficacy [1]. The complicated interplay between malignant cells and immune and stromal elements in the TME make it challenging and laborious to identify and characterize potential therapeutic targets and biomarkers from biological experiments [2]. Bioinformatics approaches capture cell type specific profiles and cell-cell interactions in vivo, and has been proven to be an effective strategy to evaluate cancer diagnoses and therapeutic responses [3].

The human guanylate-binding proteins (GBPs) family comprises 7 members that are highly responsive to IFN (interferon) stimulation [4]. GBPs have an emerging role in mediating cell-autonomous defense against intracellular pathogens by killing the bacteria directly or promoting antimicrobial signal-mediated defense [5, 6]. Among the GBPs, GBP5 specifically promoted the assembly of the NLRP3 inflammasome in response to live *L*.*monocytogenes*, *S.typhimurium* and soluble inflammasome priming agents in IFN-γ primed cells [7].

IFN-γ can be produced by immune cells, such as T cells, NK cells, and act on, both immune cells and tumor cells [8]. IFN-γ mediated surveillance is essential for eliminating or keeping malignant cells in a dormant state [9]. However, IFN-γ up-regulates PD-L1 expression, which helps cancer to escape immunological surveillance [10]. As an ISG (interferon stimulated gene), GBP5 can be inducibly increased by nearly three orders of magnitude within 24 h of IFN-γ exposure [11]. In addition, GBP5 is considered to be a useful candidate marker of the human classically activated macrophages (M1) phenotype which is differentiated with IFN-γ [12]. And conversely, GBP5 enhances the expression of virus-induced IFN, IFN-related effectors and proinflammatory factors such as IL6, IL8 and TNFα via NF-κB signaling [13]. In RA (rheumatoid arthritis), GBP5 inhibits IL1β-induced synovial inflammation and tissue destruction through modulation of cytokine-cytokine receptor interactions [14].

GBP5 has been linked to several distinct types of cancers. Truncated human GBP5 isoform lacking the C-terminal 97 amino acids (including the prenylation motif) has been associated with cutaneous T cell lymphomas [15, 16]. In addition, GBP5 is highly expressed in gastric adenocarcinomas and medullary carcinoma and promotes malignancy of GBM (glioblastoma) via the Src/ERK1/2/MMP3 pathway [17–19]. Taken together, GBP5 appears to be associated with cancer progression. However, it is unclear whether GBP5 can be used as a biomarker to predict cancer patient survival after ICB therapy. Here, we systematically analyzed the expression, molecular functions, immunological characteristics, and therapy responses of GBP5 in pan-cancers through multi-omics database mining.

## Materials and methods

### Expression for GBP5 in normal tissues and cancer tissues

Immunostaining images of GBP5 expression in human normal and cancer tissues were collected from the Human Protein Atlas [20]. The RNAseq data and relevant clinical data across 33 cancer cohorts and normal tissues of 15,776 samples were obtained from The Cancer Genome Atlas (TCGA) database of the National Cancer Institute and GTEx database by UCSC XENA [21]. R software v4.5.2 was used for statistical analysis and the ggplot2 package was used for visualization.

### Genetic alteration analysis

The cBioPortal (cBio Cancer Genomics Portal, https://www.cbioportal.org/) was utilized to investigate the mutations with GBP5 gene within diverse cancer types. We searched for “GBP5” with the option “PanCancer Studies” which contains 11 studies. Survival outcomes such as overall survival (OS) and progression free survival (PFS) were compared between GBP5-altered and unaltered tumors using cBioPortal’s “Comparison/Survival” feature.

### Comparison of the expression of GBP5 before and after IFN-γ exposure

The RNAseq data with accession number GSE154996 for human melanoma cell lines with and without IFN-γ exposure (5 ng/mL) for 6 h was from GEO (Gene Expression Omnibus) database [22]. Fifty paired data were used for analysis, and JAK1 and JAK2 knockout cells were excluded in statistical analysis.

### Correlation analysis between the expression of GBP5 and IFNG in tumor tissues

The following publicly available datasets downloaded from the GEO website were used in this study: GSE126964 (55 tumor tissues and 11 matched normal tissues from Chinese clear cell renal cell carcinoma patients), GSE82105 (total 31 samples which sampling at different time points from lesional and no lesional sites of 6 melanoma patients sensitized to 0.4% diphencyprone), and GSE149723 (total 30 samples from high grade serous ovarian cancer patients before and after a 2 week treatment with prexasertib) [18, 23, 24]. The Spearman’s test was conducted to evaluate the correlation between GBP5 and IFNG expression levels.

### Immunofluorescence

Human cell line THP-1 was purchased from American Type Culture Collection (ATCC, United States, RRID: CVCL_0006). THP-1 cells were cultured with 20 ng/mL PMA (2-acetoxy-1-methoxypropane, Cat: 16561-29-8, Meilunbio, China) for 24 hours to adhere to glass films, then the cells were left untreated or treated with LPS (Lipopolysaccharides from Escherichia coli O55:B5, 500 ng/mL, Sigma-Aldrich, USA), IFN-γ (human interferon γ, 25 ng/mL, Novoprotein, China) or IFN-γ + LPS for 16 hours. Primary antibody used for GBP5 immunofluorescence staining was from Cell Signaling Technology (Cat: #67798, USA, RRID: AB_2107436). Secondary antibody was Alexa Fluor488 conjugated goat anti-rabbit antibody (Cat# R37118, Invitrogen, USA, RRID: AB_2556546). Cell nuclei were stained with DAPI (4’,6-Diamidine-2’-phenylindole dihydrochloride, Sigma-Aldrich, USA). Images were captured with a TCS-SP8 confocal microscope (Leica, Germany). For fluorescence intensity quantification, images were analyzed by Image J.

### *GBP5* gene knockout in THP-1 cells and transcriptome analysis

Generation of *GBP5* knockout THP-1 cells and RNA sequencing were as our previously described [25–27]. Briefly, wild-type and *GBP5* gene knockout THP-1 cells were either left untreated or stimulated with IFN-γ + LPS for 16 h. Three biological replicates were included for each experimental condition. Total RNA was isolated for transcriptome sequencing, followed by clustering analysis of the differentially expressed genes.

### Single-cell analysis

Using the Cell Landscape and Human Colon Cancer Atlas (c295) to find the distribution of GBP5 in human cells and tissues, and compared the expression profiles of all 7 human GBP family members across various cell types [28]. We used single-cell expression data from CancerSEA (Cancer Single-cell State Atlas), which is the first dedicated database aiming to comprehensively decode distinct functional states of cancer cells at single-cell resolution, to explore the function of GBP5 [29].

### GO and KEGG enrichment analyses

The Gene Ontology (GO) and Kyoto Encyclopedia of Genes and Genomes (KEGG) enrichment analyses were conducted with genes strongly correlated (Spearman’s correlation coefficient > 0.6) to the expression of GBP5 from TCGA (KIRC, SKCM and UCEC) database. R software version 4.2.1 and clusterProfiler (version 4.4.4) package were used for statistical analysis and the ggplot2 (version 3.4.4) package was used for visualization [30].

### Tumor-infiltrating immune cells analysis

We used the TIMER3.0 (Tumor Immune Estimation Resource) server to analyze correlations between GBP5 expression and infiltration of immune cells (immune score) across 40 TCGA cancer types [31–33]. The correlation analysis was conducted using the purity-corrected partial Spearman’s rho value. Tumor purity: genes highly expressed in the microenvironment are expected to have negative associations with tumor purity, while the opposite is expected for genes highly expressed in the tumor cells. Heatmap was used to visualize infiltration levels of immune cells across 40 TCGA cancers.

### 10 Prediction of potential sensitivity to immune checkpoint blockades therapy

We used CIDE web server [34] (Cancer Immunology Data Engine) to perform the comparison between GBP5 and other established biomarkers on their predictive power of response outcome and overall survival in multiple cohorts. Of all studies included in the analysis, only cohorts demonstrating statistically significant differences were presented in the supplementary figures [35–49]. The established biomarkers included TIDE (Tumor Immune Dysfunction and Exclusion) score, TIDE.geneset score, TIDE.exclusion score, IMPRES (IMmuno-PREdictive Score), CytoSig.IFNG, IFNG6, IFNG, CD38, CXCL13, PDL1, CytoSig.TGFB1, TGFb, CD8, Cytolytic, Cytotoxic T, T effector, BATF3_DC, T.Clonality, mutation_burden and Aneuploidy. RECIST evaluates tumor response by measuring the sum of the longest diameters (SLD) of target lesions at baseline and during follow-up. Therapeutic response is classified as complete response (CR), partial response (PR), stable disease (SD), or progressive disease (PD) based on the percentage change in SLD relative to baseline.

## Results

### GBP5 is aberrantly expressed in the infiltrated immune cells

**Fig. 1:**
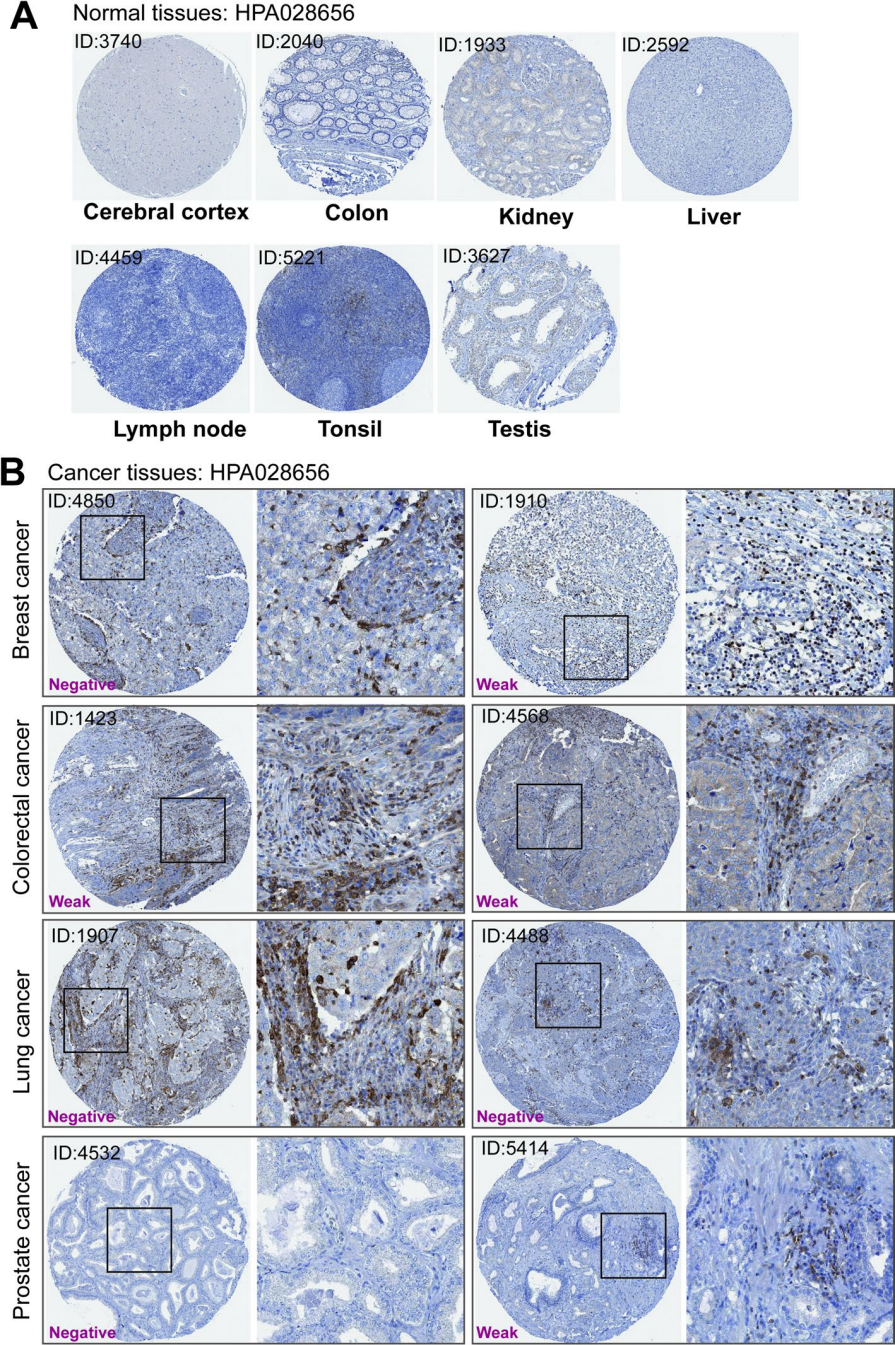
Guanylate-binding protein 5 (GBP5) expression profile in normal human tissues and different tumor types. **A** Immunohistochemical (IHC) analysis of GBP5 in normal human cerebral cortex, colon, kidney, liver, lymph node, tonsil and testis from Human Protein Atlas (HPA). The patient ID is shown at the upper left of the graph. **B** HPA immunohistochemical analysis of GBP5 in various human tumor tissues. Two patients were shown for each cancer type. The black boxed regions of GBP5 expression are shown at higher magnification at the right. The intensity for IHC staining of the tumor cells provided by HPA are indicated at the lower left corners of the images

According to the Human Protein Atlas (HPA) database, GBP5 protein was absent or expressed at very low levels in cerebral cortex, colon, kidney, liver, lymph node, tonsil and testis tissues under physiological conditions (Fig. 1A). Immunohistochemical staining from HPA revealed that GBP5 protein expression was upregulated in infiltrating cells of breast cancer, colorectal cancer, lung cancer and prostate cancer, whereas it was absent or expressed at low levels in the corresponding tumor cells (Fig. 1B).

### Single-cell transcriptome data indicated that GBP5 is mainly expressed in immune cells

**Fig. 2:**
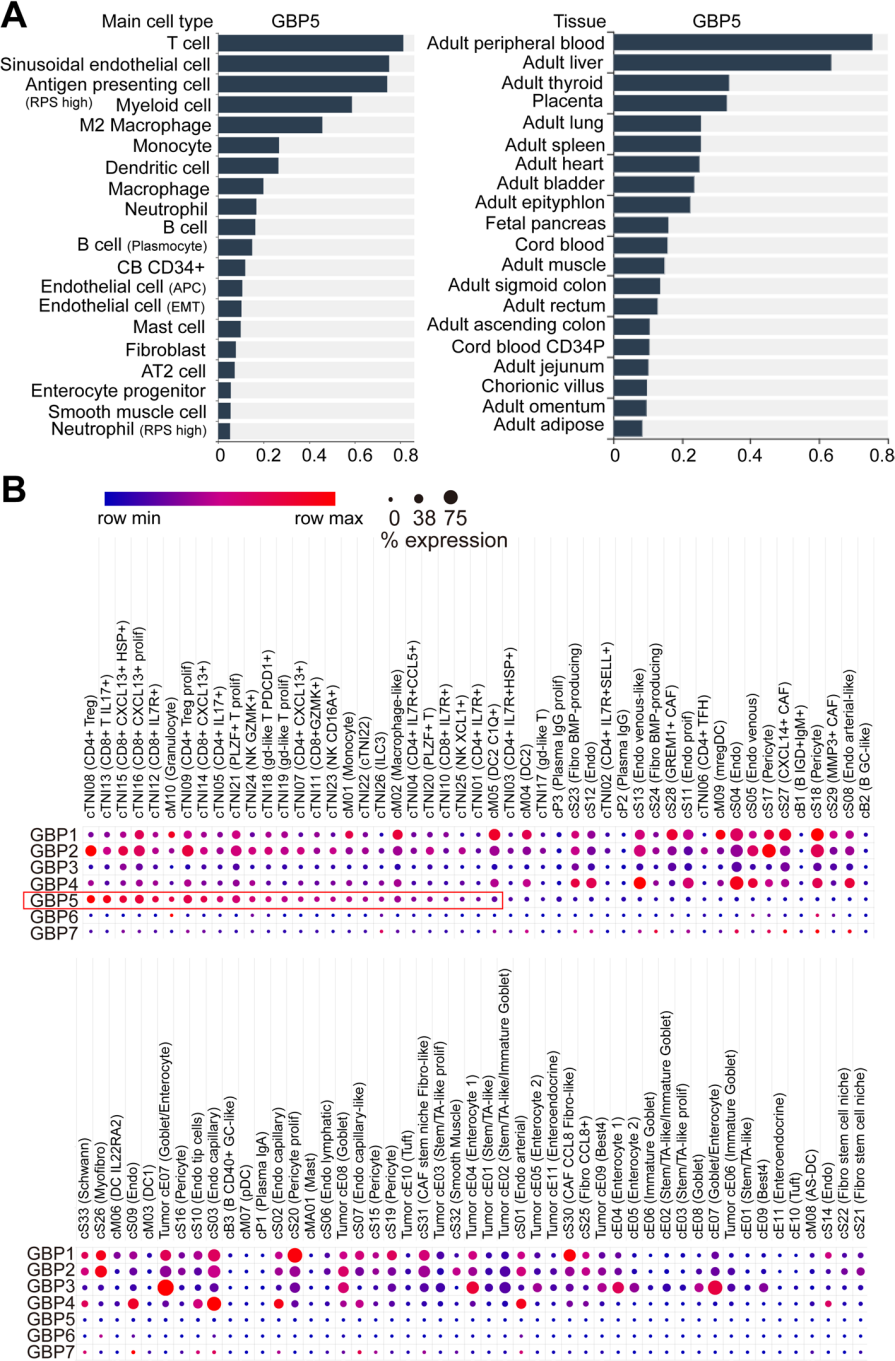
Single-cell database analyses were performed to compare the cellular expression patterns of GBP5. **A** Analysis of the cellular and tissue expression patterns of GBP5 in normal human bodies was conducted using the Cell Landscape database. **B** The cellular expression patterns of GBP1-7 were analyzed via the Human Colon Cancer Atlas database

Through Cell Landscape, we found that GBP5 is primarily expressed in cell types including T cells, sinusoidal endothelial cells, antigen-presenting cells, myeloid cells, macrophages, monocytes, dendritic cells, neutrophils, and B cells. It is simultaneously most highly expressed in peripheral blood cells and liver of adults (Fig. 2A). Using the Human Colon Cancer Atlas, we found that GBP1-5 are expressed in cell types of colorectal cancer. Among these, GBP5 exhibits more specific expression patterns compared to GBP1-4, predominantly in T cells, granulocytes, NK cells, monocytes, macrophages (Fig. 2B).

### GBP5 is highly expressed in tumor tissues compared to normal tissue across most cancer types

**Fig. 3:**
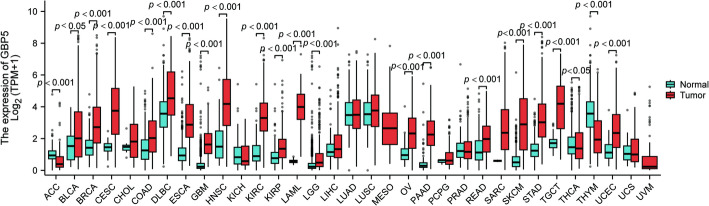
GBP5 expression levels in different tumor types from TCGA and GTEx database. TPM: gene transcripts per million reads. Statistics: Wilcoxon rank sum test

To compare the GBP5 expressions between tumors and normal tissues, we analyzed GBP5 mRNA level across 33 cancer types with corresponding normal control tissues based on TCGA and GTEx datasets. The results showed that the GBP5 expression was elevated in BLCA (bladder urothelial carcinoma), BRCA (breast invasive carcinoma), CESC (cervical and endocervical cancer), COAD (colon adenocarcinoma), DLBC (diffuse large B-cell lymphoma), ESCA (esophageal carcinoma), GBM (glioblastoma multiforme), HNSC (head and neck squamous cell carcinoma), KIRC (kidney renal clear cell carcinoma), KIRP (kidney renal papillary cell carcinoma), LAML (acute myeloid leukemia), LGG (brain lower grade glioma), OV (ovarian serous cystadenocarcinoma), PAAD (pancreatic adenocarcinoma), READ (rectum adenocarcinoma), SKCM (skin cutaneous melanoma), STAD (stomach adenocarcinoma), TGCT (testicular germ cell tumors), THCA (thyroid carcinoma), UCEC (uterine carcinosarcoma) compared to their corresponding normal control tissues. Lower expression was noticed in ACC (Adrenocortical carcinoma) and THYM (thymoma) compared to their corresponding normal controls (Fig. 3). Utilizing the cBioPortal database, we conducted an analysis of genomic alterations in GBP5 across 11 pan-cancer studies. Among 89 cancer types, genetic alteration including mutation, amplification, deep deletion and multiple alterations of GBP5 was observed in 18 cancers. The highest alteration frequency of GBP5 (11.11%) appeared in the patients with non-melanoma skin cancer. Lung cancer and medulloblastoma followed at 5.26% and 4.76%, respectively (supplementary Fig. 1A). The missense mutations are the most common genetic variation in pan-cancers, with an example in cutaneous melanoma and lentigo maligna melanoma of D411N/V mutations (supplementary Fig. 1B). The correlation analysis between mutation status of GBP5 and clinical outcomes, uncovering a positive association between GBP5 alterations and OS (*p* = 0.0335), and a negative association with PFS (*p* = 0.963) (supplementary Fig. 1C).

### Correlation of GBP5 and IFNG in cancers

**Fig. 4:**
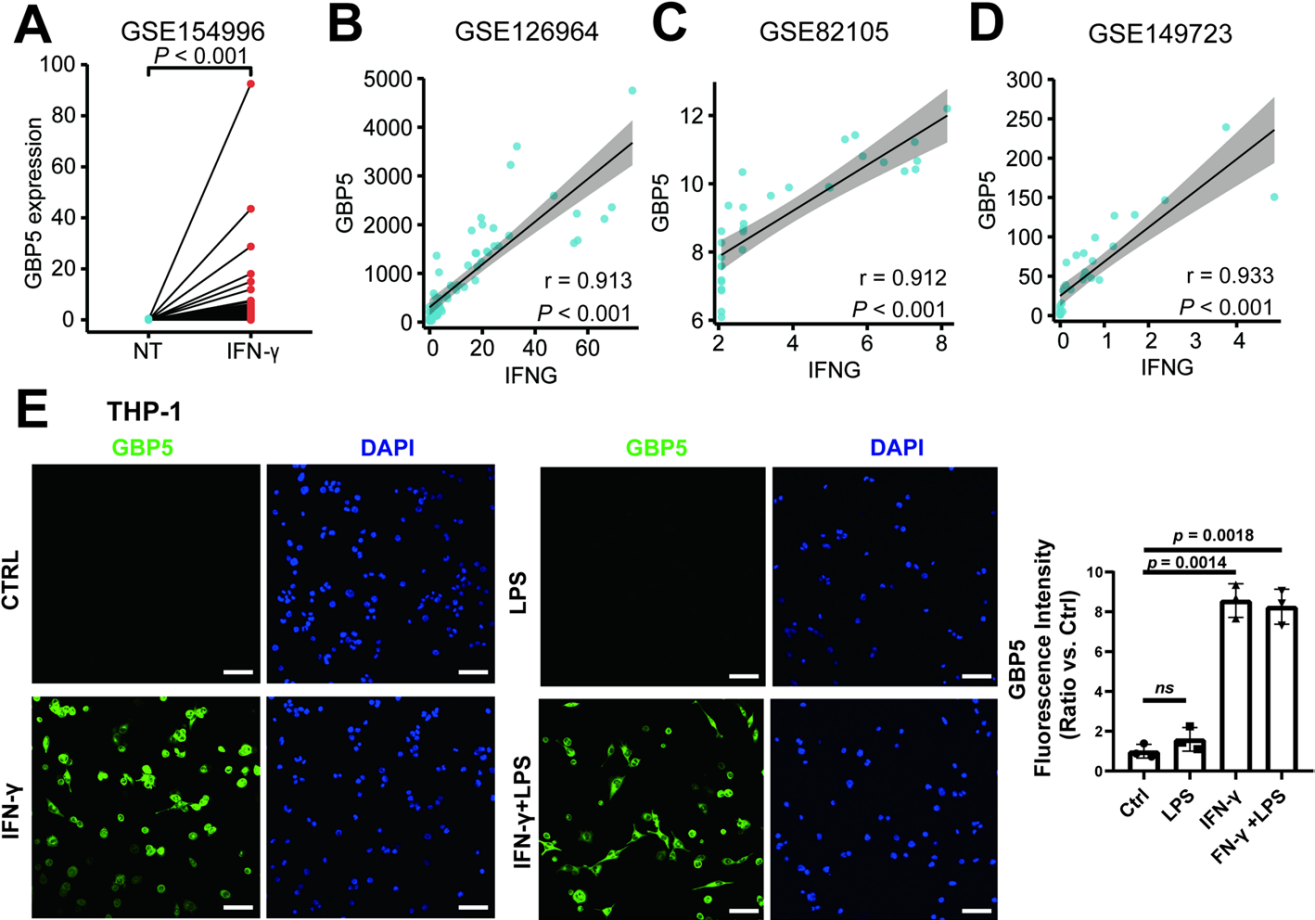
Associations of GBP5 and IFNG expression in human cancers. **A** Data were derived from GSE154996. Melanoma cell lines were left untreated (NT) or treated with IFN-γ (5 ng/mL) for 6 h. Statistics: Paired student’s *t*-test. **B-D** Spearman’s correlation analysis between GBP5 and IFNG expression in GSE126964 (B), GSE82105 (C) and GSE149723 (D). **E** Immunofluorescence staining of GBP5 (green) in THP-1 cells. Cells were untreated (control group, CTRL) or treated with LPS (500 ng/mL), IFN-γ (25 ng/mL) or IFN-γ/LPS for 16 h. Nuclei were stained with DAPI (blue). Scale bar, 75 μm. GBP5 fluorescence intensity was quantified using ImageJ software. The relative fluorescence ratio was calculated by normalizing to the mean value of the control group. Data are presented as the mean ± standard error of the mean (SEM) from three independent experiments, and statistical significance was determined using Student’s *t*-test

Consistent with that GBP5 is an interferon stimulated gene (ISG), GBP5 expression in melanoma cell lines was increased after IFN-γ stimulation (Fig. 4A). Spearman correlation analysis with three published cohorts indicated that the GBP5 expression level was strongly correlated with the IFNG expression level (Fig. 4B–D, Spearman’s rank correlation coefficient: *r* = 0.913 in GSE126964, *r* = 0.912 in GSE82105, *r* = 0.933 in GSE149723). Immunofluorescence staining of in vitro cultured THP-1 cells (human monocytes cell line that differentiates into a macrophage-like phenotype upon PMA stimulation) indicated that GBP5 expression was robustly increased after stimulation with IFN-γ. Moreover, when treated with LPS alone, GBP5 expression in THP-1 cell was not increased significantly. In contrast, when treated with IFN-γ and LPS, THP-1 cells exhibited increased expression of GBP5, and simultaneously a morphological change indicating M1 activation of the macrophage (Fig. 4E).

### GBP5 plays an important role in immune regulation across cancers

**Fig. 5:**
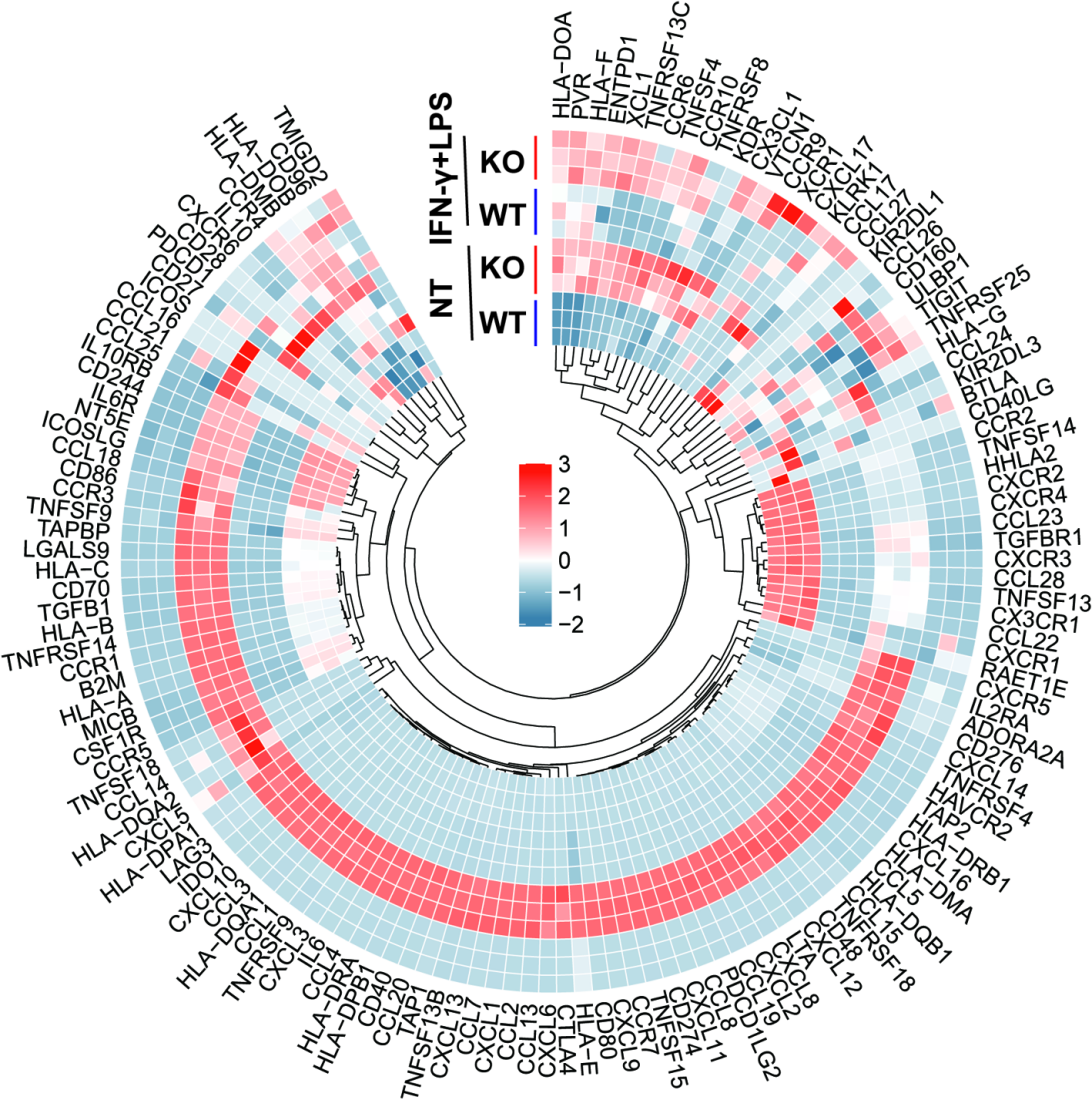
The effect of GBP5 knockout on immune-related genes in THP-1 cells. The heatmap showing the gene expression of five modules (chemokines, chemokine receptors, MHC molecules, immunoinhibitors and immunostimulators) in parental THP-1 cells and GBP5 knockout cells, untreated or treated with IFN-γ/LPS. IFN-γ/LPS stimulation was performed for 16 h using 25 ng/mL IFN-γ and 500 ng/mL LPS

To gain further insights on the potential roles of GBP5 in immune responses, we used stable knockout (KO) of the GBP5 gene in THP-1 cells. We reanalyzed our previous data to examine the expression of tumorigenesis related genes, such as MHC molecules which were not analyzed in our previously published paper.31 We performed hierarchical clustering with genes in the modules of immunoinhibitors, immunostimulators, MHC molecules, chemokines and chemokine receptors, and found that the influence of GBP5 on immunological reactions was highly extensive. Most genes examined were upregulated after IFN-γ/LPS stimulation in parental THP-1 cells, but were not changed in GBP-KO cells (Fig. 5).

### Functions of GBP5 in pan-cancers

**Fig. 6:**
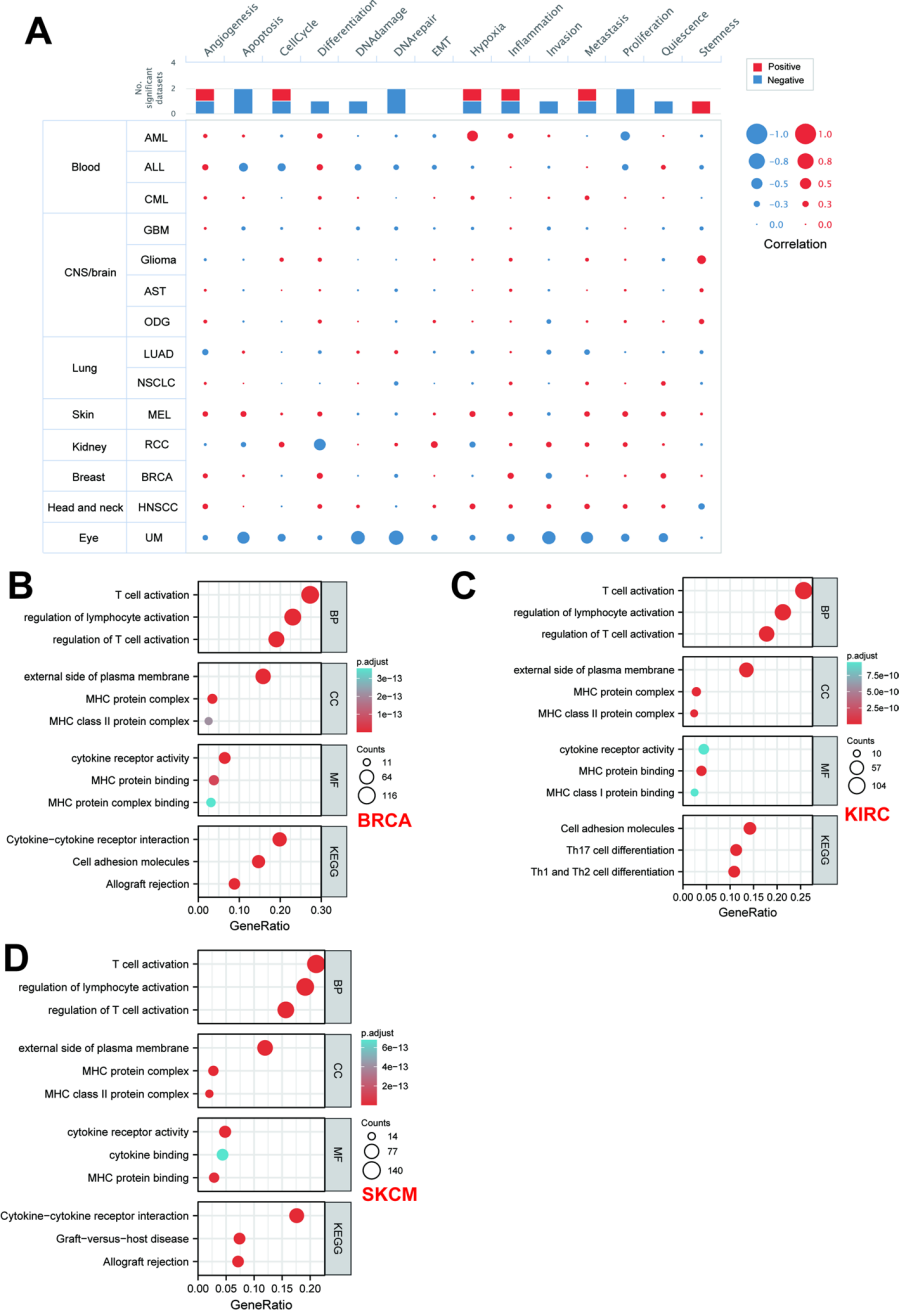
Functional analysis of GBP5 across distinct cancers. **A** Main graph: average correlations between GBP5 expression and functional modules in different cancers. The bar chart shows the number of datasets in which GBP5 was significantly correlated to the corresponding functional module. Data were derived from CancerSEA. **B-D** GO and KEGG analyses of genes strongly correlated (Spearman’s correlation coefficient > 0.6) to the expression of GBP5 in BRCA (B), KIRC (C) and SKCM (D). Data were derived from TCGA

To explore the potential functions of GBP5 in cancers, we performed single-cell analysis using CancerSEA. The results indicated that GBP5 is positively correlated with stemness, and negatively correlated with apoptosis, differentiation, DNA damage, DNA repair, invasion, proliferation and quiescence. The correlation of GBP5 with angiogenesis, cell cycle, hypoxia, inflammation and metastasis varied for different cancer types. Interestingly, GBP5 was positively correlated with inflammation in 13 distinct cancers except in UVM (Fig. 6A). Next, genes strongly correlated (Spearman’s correlation coefficient > 0.6) with GBP5 expression in BRCA, KIRC and SKCM in the TCGA database were identified and subjected to GO and KEGG enrichment analyses in three cohorts separately. The enriched terms were very similar in these three cohorts (Fig. 6B-D). Enriched Biological Process (BP) in all three cohorts included T cell activation, regulation of lymphocyte activation and regulation of T cell activation. The enriched Cellular Component (CC) common for all cohorts included external side of plasma membrane, MHCI complex and MHC II complex. The enriched Molecular Function (MF) common for all cohorts included cytokine receptor activity and MHC protein binding. For KEGG, the enriched terms were cytokine-cytokine receptor interaction, cell adhesion molecules, and allograft rejection in BRCA cohort; cell adhesion molecules, Th17 cell differentiation, Th1 and Th2 cell differentiation in KIRC cohort; cytokine-cytokine receptor interaction, graft-versus-host disease, and allograft rejection in SKCM cohort.

### Estimation of immune infiltration and biomarker on GBP5

**Fig. 7:**
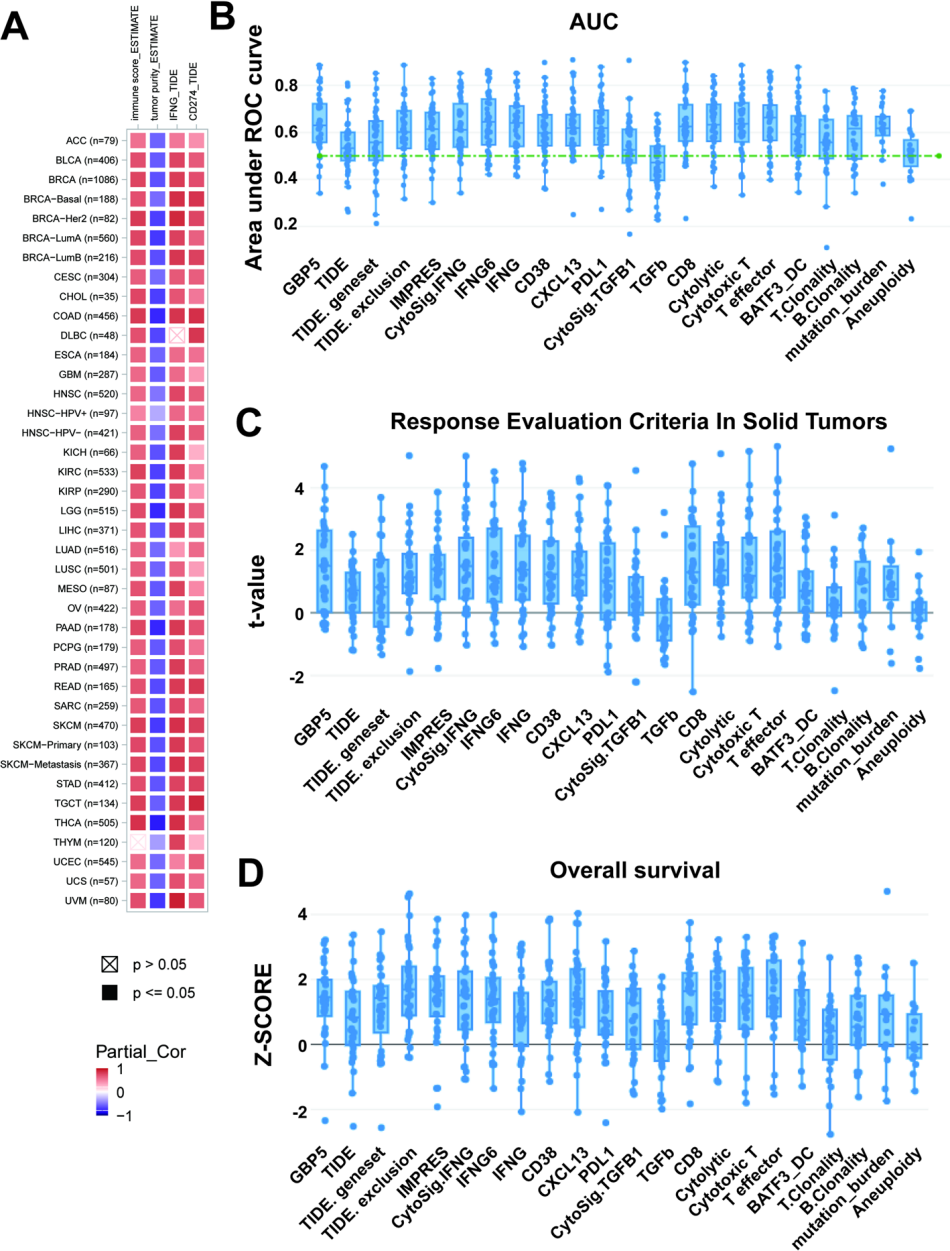
The correlation of GBP5 expression with immune infiltration level and biomarker evaluation in diverse cancer types. **A** Heatmap showing the correlations of GBP5 expression with infiltration by immune cells types in diverse TCGA cancer types. **B-D** Pooled analyses of omics datasets from immunotherapy cohorts showing the predictive performance of GBP5 on the responsiveness of ICB (**B**), RECIST (**C**) and OS (**D**) in immunotherapy sub-cohorts, compared to established cancer biomarkers from CIDE web server. The AUC was used to evaluate the predictive performances of the test biomarkers. Each dot represents an individual cohort. ICB: immune checkpoint blockade; AUC: area under the receiver operating characteristic curve; RECIST: Response Evaluation Criteria In Solid Tumors; CIDE: Cancer Immunology Data Engine

The correlation between GBP5 expression and immune infiltration across 40 TCGA cancer types was assessed using TIMER3.0 web server. Consistent with the IHC results (Fig. 1B), the negative correlation between GBP5 expression and purity in 40 cancer types indicated that GBP5 was highly expressed in the microenvironment, but not in tumor cells. GBP5 expression was positively associated with immune scores, IFNG scores and CD274 scores reinforcing its role as an effector immune marker (Fig. 7A).

Then we evaluated the capability of GBP5 expression in predicting response outcomes and OS of immunotherapy in comparison with established biomarkers. We found that GBP5 alone exhibited the highest number of immune checkpoint blockade (ICB) sub-cohorts with an area under the receiver operating characteristic curve (AUC) greater than 0.7, accounting for 15 out of 43 sub-cohorts. This was followed by IFNG6 (14/43) and then the Cytolytic (13/43). In a HeadNeck Pembrolizumab cohort, GBP5 showed a superior predictive performance (AUC = 0.886) compared with the other 21 biomarkers, including TIDE, TIDE.geneset, TIDE.exclusion, IMPRES, CytoSig.IFNG, IFNG6, IFNG, CD38, CXCL13, PDL1, CytoSig.TGFB1, TGFb, CD8, Cytolytic, Cytotoxic T, T effector, BATF3_DC, T.Clonality, mutation_burden and Aneuploidy (Fig. 7B, supplementary Table 1). To further investigate GBP5 may serve as a predictive biomarker for ICB therapy response, the Response Evaluation Criteria In Solid Tumors (RECIST) was applied to compare GBP5 with the other 21 biomarkers. Among the 35 ICB sub-cohorts, significant heterogeneity in GBP5 expression across the four RECIST-defined response categories (complete response [CR], partial response [PR], stable disease [SD], and progressive disease [PD]) was detected in 12 sub-cohorts (*P* < 0.05; t-value > 2) (Fig. 7C, supplementary Fig. 2). Of the 33 cohorts included, 11 demonstrated that high GBP5 expression was associated with prolonged overall survival, whereas only one cohort exhibited the opposite association (Fig. 7D, supplementary Fig. 3). These results suggest that GBP5 is a robust predictive biomarker for ICB therapy.

## Discussion

In this study, we demonstrated that GBP5 was closely related to the occurrence and progression of various cancers through immune responses. IHC from HPA combined with tumor purity analysis from TIMER3.0 verified that GBP5 was barely expressed in tumor cells, but predominantly expressed in infiltrated immune cells across various cancers. The immune cells in the TME include but not limited to NKs (natural killer cells), DCs (dendritic cells), macrophages, polymorphonuclear leukocytes, mast cells and CTLs (cytotoxic T lymphocytes) [50]. GBP5 is predominantly expressed in immune-related cell types and exhibits more specific expression in colorectal cancer associated immune cells than other 6 GBPs, as revealed by Cell Landscape and the Human Colon Cancer Atlas. Cytokines which can be generated from all the cell types, are important non-cellular components of TME [51]. We confirmed that IFN-γ promoted the expression of GBP5 in macrophages, and in clinical treatment, there was a strong correlation between the expression of GBP5 and IFN-γ. Our results revealed low GBP5 expression in tumor cells, however, existing study has demonstrated that IFN-γ can also induce the upregulation of GBP5 in tumor cells, and high GBP5 expression in tumor cells can modulate the proliferation and metastasis of the tumor cells themselves [17, 52]. In addition to IFN-γ, GBP5 can also be induced by several other cytokines, such as IL18, TNFα, IFN-α14 and IFN-α2 [14, 53]. Here our study is limited in that the GBP5 expressing cells were determined to be immune cells solely based on their typical morphologies. Further studies are needed to gain more accurate knowledge on the cellular distribution of GBP5.

In this study, we found that the mRNA level of GBP5 was upregulated in the tumor tissues compared to normal tissues in 20 out of 33 cancer types, and decreased in only 2 cancer types. This speculation was consistent with the results of single cell analysis with CancerSEA. In cancers, acute inflammatory responses help kill and clear cancer cells, while chronic inflammation promotes carcinogenesis and tumor development [54, 55]. Our results showed that GBP5 promoted inflammation in most cancer types. In addition, GBP5 is involved in angiogenesis, apoptosis, cell cycle, differentiation, DNA damage, DNA repair, EMT, hypoxia, invasion metastasis, proliferation, quiescence and stemness, but behaved differently in different cancer types. Overall, GBP5 expression is correlated with stronger antitumor activity in most cases, however there are some exceptions due to the heterogeneity of tumors. The inherent heterogeneity and complexity of cancer result in unique biological profiles for each malignancy, along with distinct hurdles to its clinical treatment [56]. Therefore, the characteristics of GBP5 identified in our pan-cancer analysis may not be applicable to one specific type of malignancy, which necessitates further investigation using clinical samples and experimental studies. Given that GBP5 is inducibly activated by IFN-γ, GBP5 may serve as a context-dependent biomarker, particularly in IFN-γ-driven tumors such as melanoma and bladder cancer.

Previous study shows that THP-1 cells can be induced into classically activated macrophage-like cells (M1) by IFN-γ and LPS [57]. After stimulation with IFN-γ and LPS, THP-1 cells, most of the immune response molecules were up-regulated, while the sensitization was significantly weakened in GBP5 knockout cells. These data indicated that GBP5 was not a byproduct of excessive cytokines, but actively participated in immunoregulation in tumors. GBP5 was reported to play a role in immunoreactive microenvironment in NSCLC (non-small cell lung cancer), oral squamous cell carcinoma, ovarian cancer, and clear cell renal cell carcinoma [58–61]. Similar results were also obtained in the immunoinfiltration analysis with TIMER3.0 in our study, which showed significant positive correlations of GBP5 expression with infiltration immune cells in 40 cancer types. At the molecular and pathway levels, GO and KEGG analysis revealed that genes strongly associated with GBP5 were enriched in lymphocytes, MHC and cytokine related pathways. Taken together, as a molecule which highly expressed in only tumor infiltration immune cells (TIICs), GBP5 affects the crosstalk between tumor cells and TIICs through regulating cytokines, cytokine receptors, and chemokines.

As outcome of cancer depends largely on the crosstalk between the tumor and its microenvironment, in recent years, clinical trials targeting immune checkpoints significantly improve survival of tumor patients [62]. Immune checkpoint blockade (ICB) with antibodies against CTLA-4/B7 and PD-1/PD-L1 showed remarkable clinical effectiveness in a variety of cancers [63], however, ICB response prediction remains an open question. A study has showed the panel of CXCL9, GBP5, and IFNG is can potentially be a biomarker for predicting immunotherapy response in different cancers [64]. However, through analysis of ICB cohorts on the CIDE web server, we found that GBP5 achieved an AUC > 0.7 in 15 of 43 ICB cohorts, and its expression was significantly higher in responsive groups across 12 of 35 cohorts. These results indicate that GBP5 may have the potential to predict the efficacy of immunotherapy in hepatocellular carcinoma, melanoma, urothelial carcinoma, metastatic renal cell carcinoma, NSCLC and relapsed small cell lung cancer. We speculate that as a cytokine inducible intracellular protein, GBP5 can sensitively monitor the “stress” in the tumor immune microenvironment, and thus to achieve a satisfactory performance in the predictions of therapeutic efficacy. While GBP5 demonstrates promising predictive efficacy for immunotherapy responses in multiple tumor types, our findings are unadjusted for multiple confounding factors, a limitation attributable to the inherent complexity of the tumor immune microenvironment. Consequently, these data are for reference purposes only, and further cross-analyses integrating a comprehensive panel of parameters in distinct cancer types are warranted to generate more robust and clinically actionable conclusions. In the analysis of the association between GBP5 and overall survival, 11 out of 33 cohorts demonstrated that high GBP5 expression was associated with a better prognosis, which indicate that GBP5 expression levels can be used for prognostic assessment in advanced clear cell renal cell carcinoma, hepatocellular carcinoma, melanoma, urothelial carcinoma, NSCLC and relapsed small cell lung cancer. While GBP5 holds promise as a prognostic biomarker in certain cancer types, its clinical potential is constrained by multiple factors, including cancer-type specificity, genetic alterations, technical variability in analytical datasets, and functional overlap with other established biomarkers. Further investigations encompassing external validation and mechanistic exploration of its roles in tumor-immune crosstalk are essential to comprehensively evaluate the clinical utility of GBP5 in clinical practice.

## Conclusion

In summary, our results suggest that GBP5 is predominantly expressed in the tumor infiltrated immune cells and plays pivotal regulatory roles in the immune-oncology context of the TME in various cancer types. Importantly, we demonstrated that GBP5 is a potential predictor for ICB efficacy. The specific biological and immunological mechanisms of GBP5 in distinct cancers warrant further studies.

## Limitation

The analyses in this study were based on data retrieved from public databases, which may differ in data collection, processing, and analytical pipelines. Such inherent heterogeneities could potentially compromise the comparability of datasets and the interpretation of the observed results. Although our study identified significant correlations between GBP5 expression and multiple cancer-related parameters, it lacked experimental validation through in vitro or in vivo models. The exact molecular mechanisms by which GBP5 modulates immune cell function to affect tumor growth and metastasis require further experimental validation. Given the retrospective and observational nature of the study design, causal inferences cannot be definitively established. The sample sizes for certain cancer types were relatively limited, which may restrict the generalizability of the findings. Additionally, the potential of GBP5 as a therapeutic target remains to be validated in preclinical models or clinical settings.

## Supplementary Information

Below is the link to the electronic supplementary material.

Supplementary Material 1.

Supplementary Material 2.

## Data Availability

Publicly available datasets were analyzed in this study. IHC images of GBP5 protein expression were downloaded from the Human Protein Atlas (HPA, https://www.proteinatlas.org/search/GBP5). The distribution of GBP5 positive cells were showed by using Human Colon Cancer Atlas (c295) of Single Cell Portal (https://singlecell.broadinstitute.org/single_cell/study/SCP1162/human-colon-cancer-atlas-c295). The expression of GBP5 in different cancers and normal tissues of 15776 samples were obtained from The Cancer Genome Atlas (TCGA) database of the National Cancer Institute (https://portal.gdc.cancer.gov) and GTEx database by UCSC XENA (https://xenabrowser.net/datapages/), followed by the extraction of expression data for the GBP5 gene across various samples in TPM format. The RNA sequencing data which performed on GBP5 knockout THP-1 cells was deposited in National Omics Data Encyclopedia (https://www.biosino.org/node/browse? keyword=OEP002938). The dataset GSE154996, GSE126964, GSE82105, and GSE149723 are available in the Gene Expression Omnibus (GEO) datasets (https://www.ncbi.nlm.nih.gov/geo/query/acc.cgi? acc=GSE154996, https://www.ncbi.nlm.nih.gov/geo/query/acc.cgi? acc=GSE126964, https://www.ncbi.nlm.nih.gov/geo/query/acc.cgi? acc=GSE82105, https://www.ncbi.nlm.nih.gov/geo/query/acc.cgi? acc=GSE149723).The immune infiltration data and immunotherapy data were derived from TIMER3.0 (https://compbio.cn/timer3/) and CIDE (https://cide.ccr.cancer.gov/), respectively.
