# Supplementary material for: Aberrant expression of GBP5 in tumor-infiltrating immune cells as an onco-immunological biomarker in pan-cancer
Source: Discov Oncol. 2026 May 27;17:1073. doi: 10.1007/s12672-026-05305-5 (PMC13396069; doi:10.1007/s12672-026-05305-5)
Supplement: Supplementary file 1 — Supplementary Material 1. [file 12672_2026_5305_MOESM1_ESM.docx]

**Supplementary Figures**


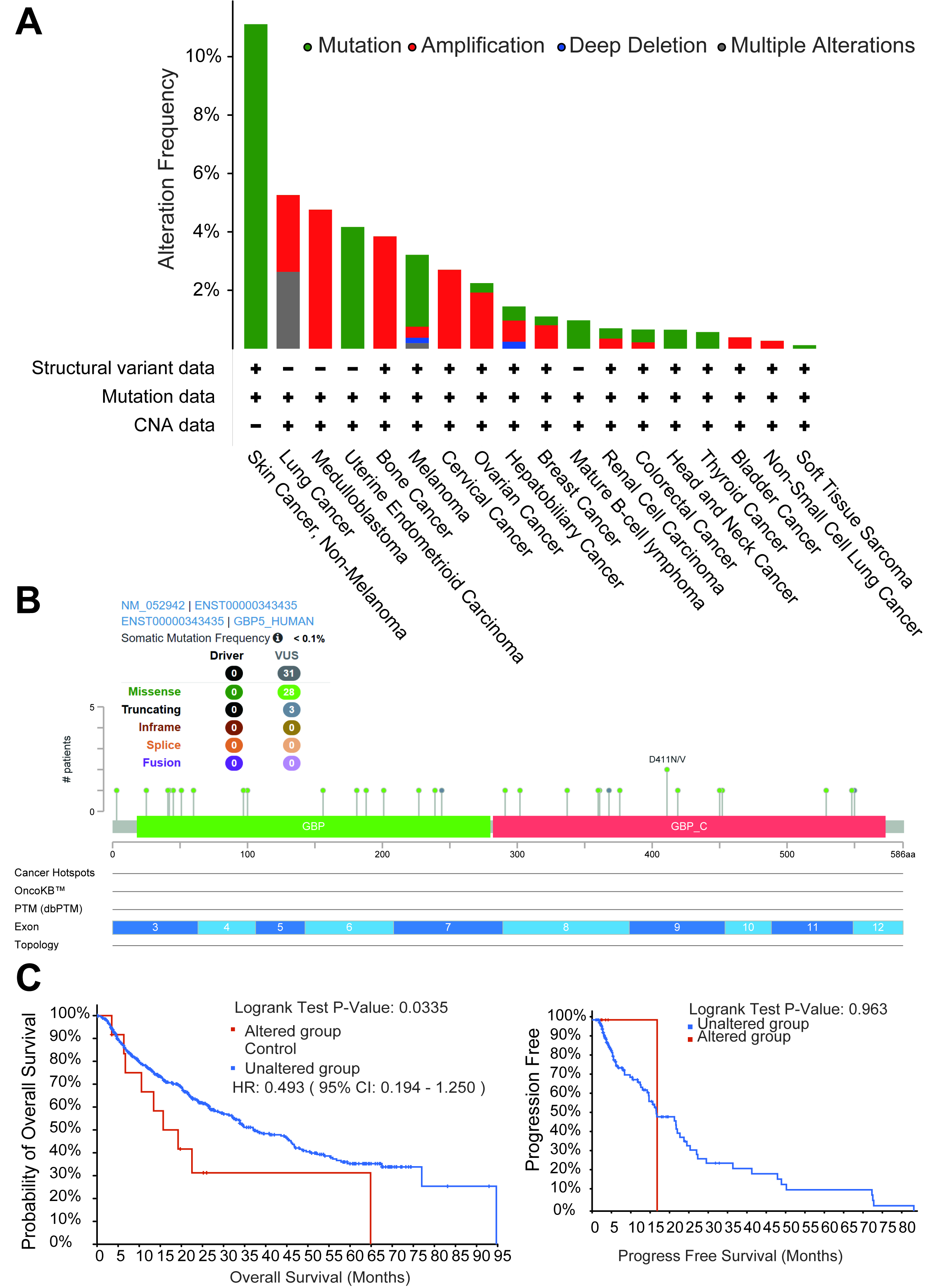


**Supplementary Fig. 1** Characterization of GBP5 mutations across TCGA tumors with cBioPortal. **A** The alteration frequency with mutation types of GBP5 are plotted. **B** The frequency of mutations at specific sites was displayed to provide detailed insights into affected loci. **C** Correlations between GBP5 mutation status and overall survival and progression free survival in pan-cancer studies.


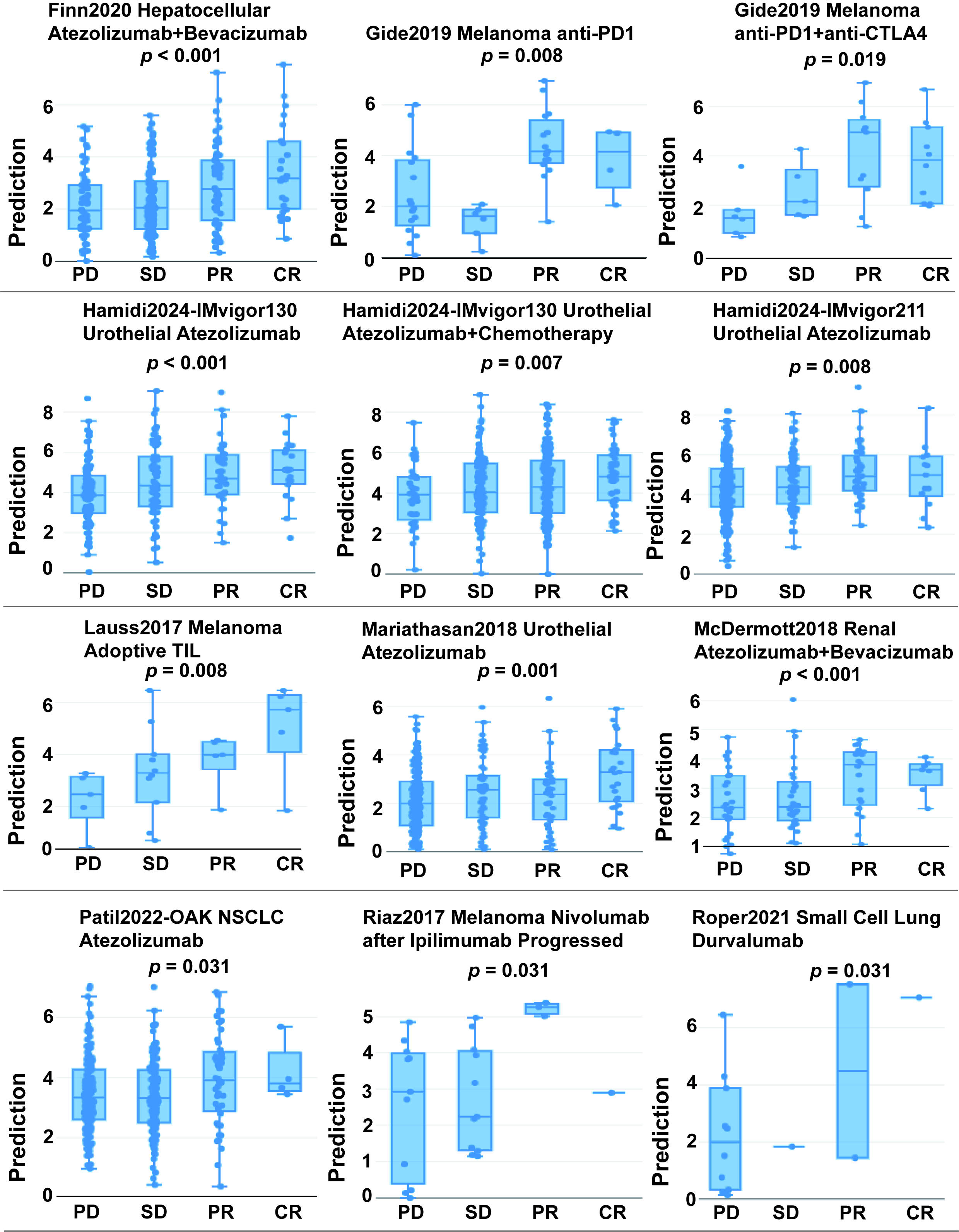


**Supplementary Fig. 2** The GBP5 expression in the 12 ICB sub-cohorts across the four RECIST-defined response categories derived from CIDE web server. CR: complete respons; PR: partial response; SD: stable disease; PD: progressive disease. RECIST: Evaluation Criteria In Solid Tumors; CIDE: Cancer Immunology Data Engine.


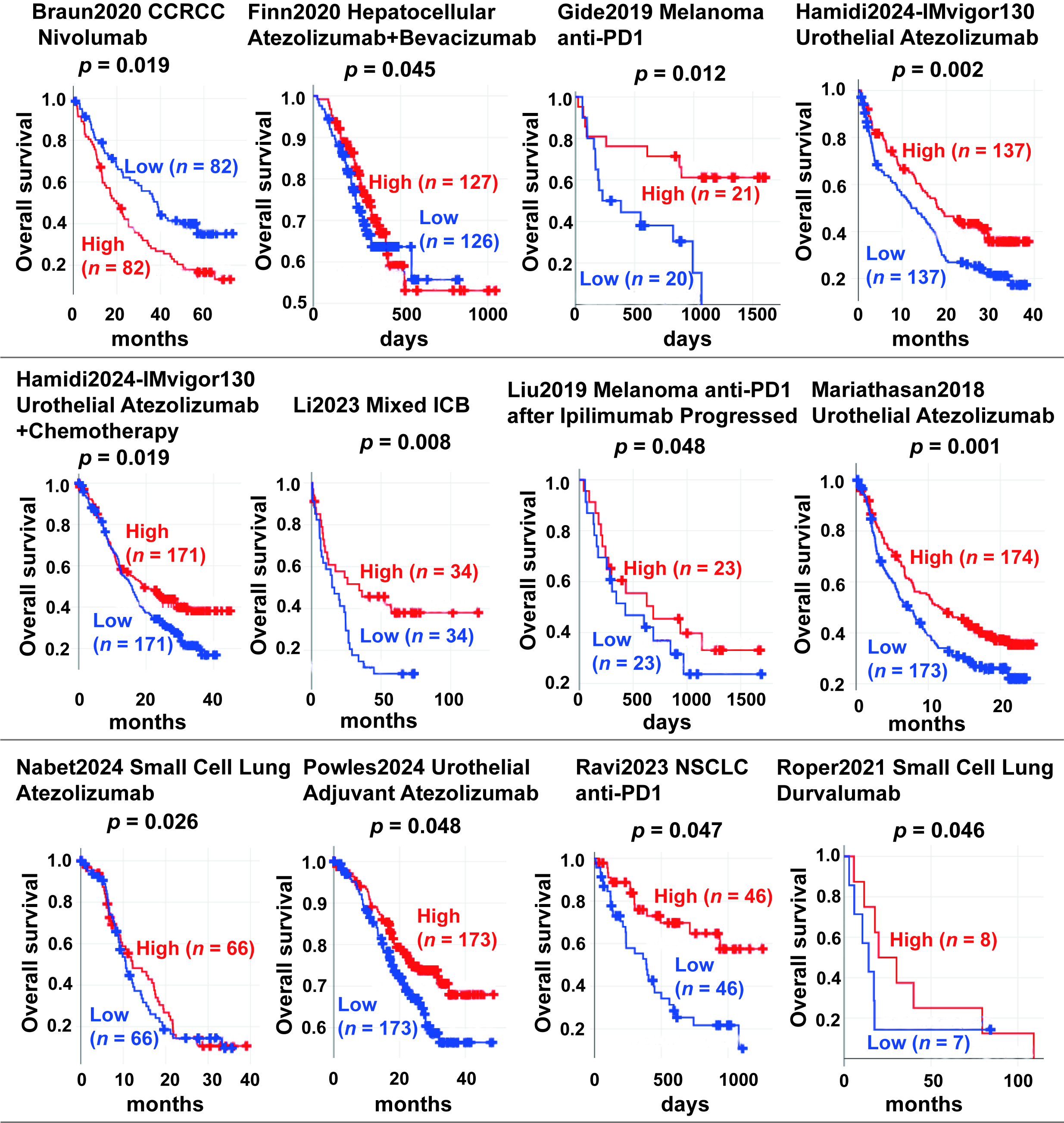


**Supplementary Fig. 3** The association of GBP5 with overall survival in 12 ICB cohorts derived from CIDE web server.
